# Mesenchymal stem cells promote type 2 macrophage polarization to ameliorate the myocardial injury caused by diabetic cardiomyopathy

**DOI:** 10.1186/s12967-019-1999-8

**Published:** 2019-08-05

**Authors:** Liyuan Jin, Zihui Deng, Jinying Zhang, Chen Yang, Jiejie Liu, Weidong Han, Ping Ye, Yiling Si, Guanghui Chen

**Affiliations:** 1 Chinese People’s Liberation Army Medical School, Chinese PLA General Hospital, No. 28 Fuxing Road, Beijing, 100853 China; 2 Department of Geriatric Cardiology, Chinese PLA General Hospital, No. 28, Fuxing Road, Beijing, 100853 China; 3 Institute of Basic Medicine Science, Chinese PLA General Hospital, No. 28 Fuxing Road, Beijing, 100853 China; 4 Department of Cardiology, Chinese PLA General Hospital, No. 28 Fuxing Road, Beijing, 100853 China

**Keywords:** Diabetic cardiomyopathy, Mesenchymal stem cell, Macrophage polarization

## Abstract

**Background:**

Diabetic cardiomyopathy (DCM) is a common complication of diabetes and is characterized by chronic myocardial inflammation. Mesenchymal stem cell (MSC) infusions have recently been suggested to alleviate myocardial injury and ameliorate cardiac function. However, few studies have focused on the effects of MSCs in DCM. Therefore, we explored the effects of MSC-regulated macrophage polarization on myocardial repair in DCM.

**Methods:**

A DCM rat model was induced by a high-fat diet and streptozotocin (STZ) administration and infused 4 times with MSCs. Rat blood and heart tissue were analyzed for blood glucose levels, lipid levels, echocardiography, histopathology, macrophage phenotype ratios and inflammatory cytokines, respectively. We mimicked chronic inflammation in vitro by inducing peritoneal macrophages with high glucose and LPS, then cocultured these macrophages with MSCs to explore the specific mechanism of MSCs on macrophage polarization.

**Results:**

DCM rats exhibited abnormal blood glucose levels and lipid metabolism, cardiac inflammation and dysfunction. MSC infusion ameliorated metabolic abnormalities and preserved cardiac structure and function in DCM rats. Moreover, MSC infusion significantly increased the M2 phenotype macrophages and alleviated cardiac inflammation. Interestingly, this in vitro study revealed that the MSCs pretreated with a COX-2 inhibitor had little effect on M2 macrophage polarization, but this phenomenon could be reversed by adding prostaglandin E2 (PGE2).

**Conclusions:**

Our results suggested that MSC infusions can protect against cardiac injury in DCM rats. The underlying mechanisms may include MSC-enhanced M2 macrophage polarization via the COX-2-PGE2 pathway.

## Background

Diabetic cardiomyopathy (DCM), one of the most serious complications of diabetes, is characterized by high morbidity and mortality. DCM pathogenesis is mainly characterized by chronic uncontrolled hyperglycemia, insulin resistance, and chronic inflammation [1]. These characteristics are further exacerbated by oxidative stress and metabolism-associated inflammation, resulting in cardiomyocyte apoptosis and interstitial fibrosis and ultimately resulting in irreversible cardiac function and even death [2, 3].

Macrophage-mediated regulation of chronic inflammation plays a key role in aggravating and ameliorating DCM. Different macrophage phenotypes play distinct roles in inflammatory diseases. M2 macrophages, a class of anti-inflammatory cells, may promote restoring injured tissues by secreting anti-inflammatory cytokines [4, 5]. Thus, increasing the number or proportion of M2 macrophages may alleviate the inflammatory response and ameliorate cardiac injury.

Mesenchymal stem cells (MSCs) appear to hold great promise in treating immunological, inflammatory and metabolic disorders [6]. Our previous study demonstrated that MSCs affect macrophage differentiation in insulin-sensitive organs and ameliorate insulin resistance [7, 8]. However, few studies have reported the effects of MSCs involved in macrophage polarization in DCM. Therefore, our study established a rat DCM model to demonstrate the therapeutic effect of MSCs and further explore the underlying mechanisms of macrophage polarization in vivo and in vitro.

## Materials and methods

### Cell cultures

#### Isolating, culturing and identifying adipose-derived MSCs

To isolate adipose-derived (AD)-MSCs, inguinal fat was obtained from Sprague–Dawley (SD) rats weighing 80–100 g. Adipose-derived-MSCs were isolated, purified, and identified as described previously [9], cultured in either a lipid-inducing solution (Cyagen) or osteogenic-inducing fluid (Cyagen) for 2 weeks, then stained with Oil Red O (Sigma) and Alizarin red (Sigma), respectively. AD-MSCs were passaged 3 to 4 times and identified by positive staining with antibodies against surface CD29 (APC, eBioscience) and CD90 (APC, BD) and negative staining with antibodies against CD34-FITC, CD45-FITC, CD11b-FITC (BD) by flow cytometry. The target cells we chose were in the FSC-Height and SSC-Height quadrant to separate cell debris.

#### Isolating, culturing and identifying peritoneal macrophages

As described previously [10], peritoneal lavage was used to obtain peritoneal macrophages. The cells were cultured with Dulbecco’s Modified Eagle’s Medium–high glucose (DMEM-HG; HyClone) complete medium containing 10% fetal bovine serum (FBS), 100 U/mL penicillin and 100 g/mL streptomycin. The medium was changed after 2 h, and the macrophages were cultured for 48 h, then stained with CD11b-FITC (BD) and Alexa Fluor 647-CD68 (AbD Serotec) antibodies to identify the peritoneal macrophage’s purity by flow cytometry.

### Establishment of a DCM rat model

Thirty 8-week-old male Sprague–Dawley (SD) rats were purchased from the Chinese People’s Liberation Army (PLA) General Hospital and fed either a high-fat diet (HFD) or a normal chow diet (NCD) for 8 weeks. To establish the DCM rat model, a single intraperitoneal injection of streptozotocin (STZ, 25 mg/kg, Sigma-Aldrich) was administered to the HFD-fed rats. Then rats of all groups were fed a normal chow diet until the end of experiment. The DCM rat model was evaluated and confirmed by echocardiography.

### AD-MSC administration

MSCs between passage 3 and 4 were used for tail-vein infusion. MSC infusion (2 × 10^6^ MSCs suspended in 0.3 mL sterile physiological saline) was performed every 2 weeks, 4 times in all. The normal rats and DCM rats were infused with 0.3 mL sterile physiological saline.

### Metabolic measurements in the rats

Rats were weighed at baseline, 8 weeks, 12 weeks, 20 weeks and calculated ratio of heart weight to body weight at the end of the experiment. Blood was collected from the tail vein at baseline, 8 weeks, 12 weeks, and 19 weeks within 24–48 h after MSC infusion. Fasting blood glucose (FBG), fasting insulin (FINS), insulin sensitivity index (ISI), total cholesterol (TC), triglyceride (TG) and intraperitoneal glucose tolerance test (IPGTT) levels were determined by ELISA or biochemical analyzer at different time points throughout the experiment. To perform the IPGTT, rats from each group were fasted for approximately 6 h and injected intraperitoneally with glucose (2 g/kg). Tail-vein blood samples were collected before injection and 30, 60 and 120 min after injection to measure the glucose levels. Data are presented as the mean ± SD. Differences between two groups were analyzed using analysis of Independent-Sample T Test. Differences between multiple groups were analyzed using analysis of variance (ANOVA).

### Echocardiography

Rats were anesthetized with isoflurane, and echocardiography was performed using a Vevo 770 ultrasound system. Left ventricular end-diastolic internal diameters (LVIDD), LV end-systolic internal diameters (LVIDS), left ventricular end-diastolic anterior wall (LVAWD), left ventricular end-systolic anterior wall (LVAWS), left ventricular end-diastolic posterior wall thickness (LVPWD), and left ventricular end-systolic posterior wall thickness (LVPWS) were measured by parasternal short axis scans at the papillary muscle level, and the fractional shortening (FS) was calculated simultaneously. The left ventricular ejection fraction (LVEF) was measured by parasternal long axis scans. Pulsed-wave Doppler imaging and tissue Doppler were used to assess early to late diastolic transmitral flow velocity (E/A) ratio and early to late diastolic mitral annular velocity (E’/A’) ratio, respectively.

### Histopathology staining

Rat hearts were isolated, imaged, weighed and measured. Heart samples from each group were fixed in 10% formalin after washing with phosphate-buffered saline (PBS), processed, embedded in paraffin, and cut into 4-μm sections at the papillary muscle level. Heart sections were evaluated by hematoxylin–eosin (H&E) and Masson’s trichrome staining. Five randomly selected photomicrographs of cardiomyocyte and cardiac fibrosis were chose for quantitative microscopic analysis, respectively. Myocytes with round nuclei and clearly defined sarcolemmal borders were selected for analysis of myocyte cross-sectional area (CSA) in H&E staining. And fibrosis was determined on the stained heart tissue sections highlighting the collagen fibers blue in Masson’s trichrome staining. Image J software was used to analyze myocyte CSA and quantification of myocardial fibrosis. Data are presented as the mean ± SD. Differences between multiple groups were analyzed using analysis of variance (ANOVA).

### In vitro experiments

Peritoneal macrophages were obtained as previously described [10]. Then, macrophages were cultured in a 12-well culture plate, and after a 2-h incubation, the culture medium was replaced with a high-glucose medium (33 mmol/L) containing 1 μg/mL LPS and incubated for 12 h. MSCs were then cocultured with treated macrophages at a ratio of 1:10 MSC:macrophage for another 48 h in a Transwell system (Corning). For the in vitro experiments, the cells were divided into the following groups: (1) normal, (2) damage group (high glucose + LPS), (3) damage + MSCs, (4) damage + MSCs + COX-2 inhibitor (10 μM, Abcam), and (5) damage + MSCs + COX-2 inhibitor + prostaglandin E2 (PGE2; 0.1 μM, Sigma). For groups 4 and 5, MSCs were pretreated with COX-2 inhibitor for 2 h before coculturing with macrophages either with or without adding PGE2 to the coculture system.

### Flow cytometry analysis

For the flow cytometry analysis, heart tissue was digested by type II collagenase (Sigma) at 37 °C for 40 to 50 min while rocking. Next, 10% FBS was added to terminate enzyme activity. The cell suspension was filtered through a 75 µm cell strainer and centrifuged at 1500 rpm for 10 min. For the in vitro experiments, macrophages from different treatment groups were collected using sterile PBS. Cells from the heart tissue or the in vitro experiment were incubated with antibodies against CD68 (Alexa Fluor 647, AbD Serotec), CD11c (FITC, AbD Serotec) and CD163 (RPE, AbD Serotec) at room temperature and kept in the dark for 20 min. Cells were then washed with PBS and analyzed using a FACSCalibur flow cytometer.

### Enzyme-linked immunosorbent assay (ELISA)

Heart tissue was weighed and homogenized in ice-cold physiological saline at a 1:9 ratio (weight/volume). The homogenate was then centrifuged at 3000 rpm at 4 °C for 10 min, and the supernatant was collected for analysis. Total protein was quantified via Coomassie blue staining. The concentrations of IL-6, IL-10, TNF- α and PGE2 in the cell culture, serum and heart tissue were detected by ELISA following the manufacturer’s instructions (R&D).

### Statistical analysis

Data are presented as the mean ± SD and were analyzed using SPSS 17.0. Differences between two groups were analyzed using analysis of Independent-Sample T Test. Differences between multiple groups were analyzed using analysis of variance (ANOVA). *P *< *0.05* was considered statistically significant.

## Results

### Characterization of MSCs and peritoneal macrophages

**Fig. 1:**
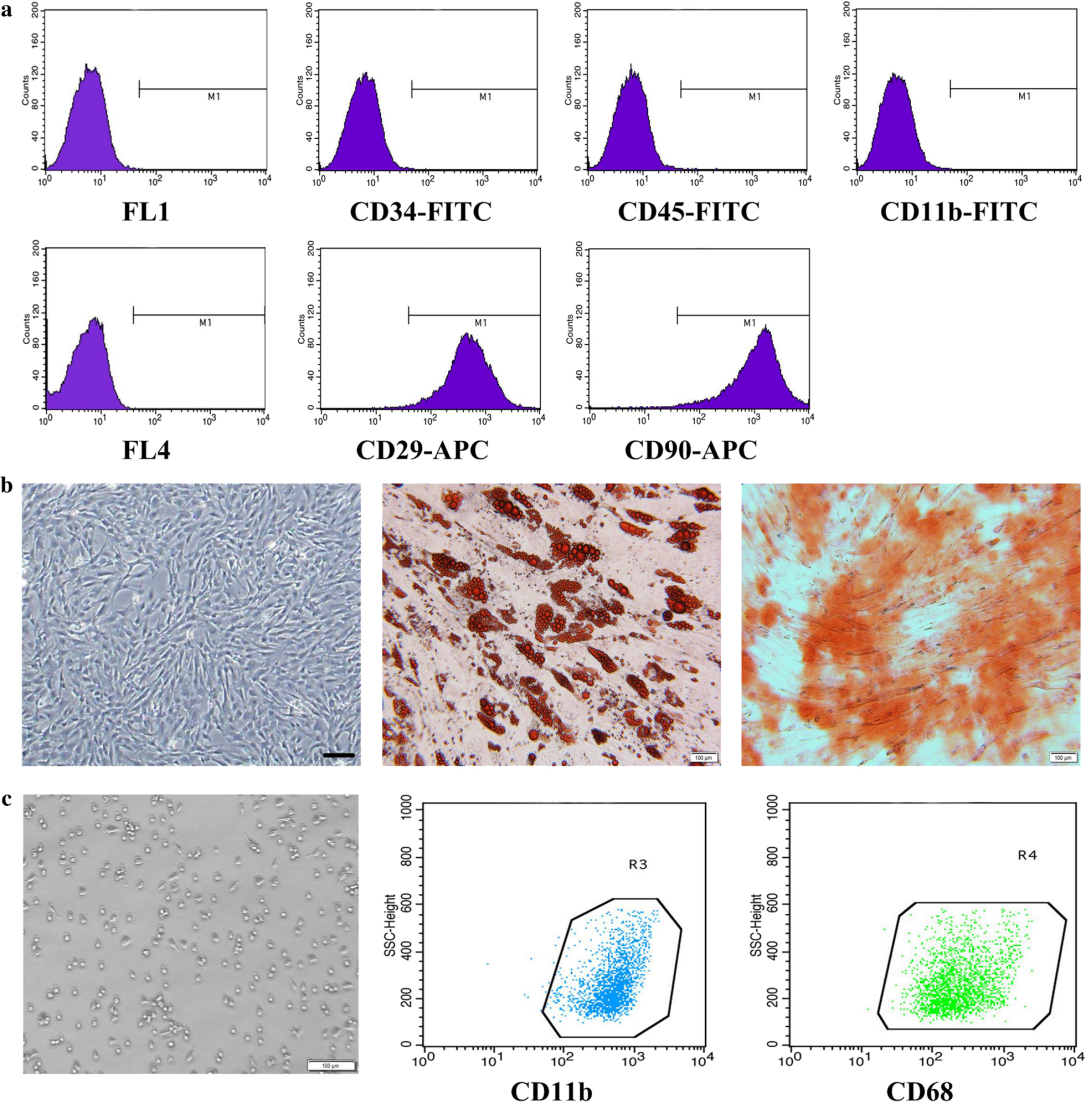
Identification of adipose-derived MSCs and peritoneal macrophages. Cultivated Passage 3 MSCs stained positive for CD29-APC and CD90-APC and negative for CD34-FITC, CD45-FITC and CD11b-FITC via flow cytometry. Negative control was also presented. The target cells we chose were in the FSC-Height and SSC-Height quadrant to separate cell debris (**a**). MSC morphology was observed via light microscopy, and MSCs were stained by Oil Red O or Alizarin Red to indicate differentiation into adipocytes or osteoblasts (**b**). Macrophage morphology was observed via light microscopy, and more than 99% of the cultured macrophages stained positive for CD11b and CD68 via flow cytometry (**c**). Scale bar: 100 μm

AD-MSCs were characterized based on their morphology, phenotype and differentiation capacity after three passages. Flow cytometry showed that more than 90% of these cells were positive for CD29 and CD90 but negative for CD34, CD45, and CD11b (Fig. 1a). The AD-MSC morphology exhibited a typical spindle-like shape and formed a swirl-like monolayer. AD-MSCs cultured in lipid-inducing solution showed many lipid droplets after Oil Red O staining, and cells cultured with osteogenic-inducing fluid stained orange (Fig. 1b).

Macrophages were also characterized based on morphology and phenotype. Figure 1c showed that the macrophages were highly refractive, large and round. Flow cytometry results showed a highly pure cell population (> 99%) positive for specific surface antibodies CD11b and CD68 (Fig. 1c).

### Characteristics of the rat diabetic cardiomyopathy model

**Fig. 2:**
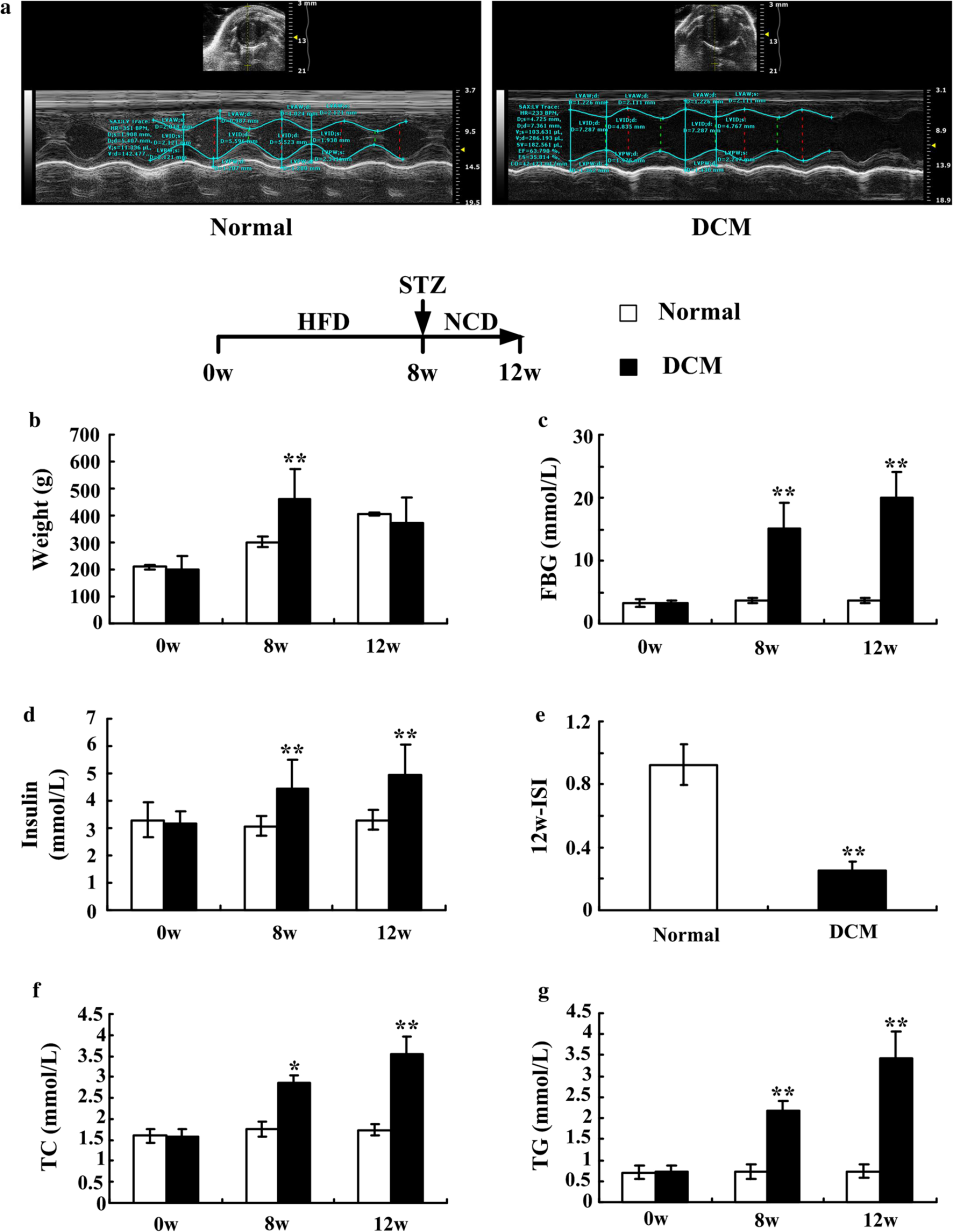
Characterization of a diabetic cardiomyopathy rat model. Rats were fed either a normal or HFD for 8 weeks and DCM rats were injected intraperitoneally with STZ (25 mg/kg). After that, all rats fed a normal chow diet (NCD). Echocardiography was performed on the rats 4 weeks after STZ injection (**a**). Body weight (**b**), fasting blood glucose (**c**), and fasting serum insulin (**d**) were assessed at baseline, 8 weeks and 12 weeks, and the insulin sensitivity index (**e**) was calculated by 1/FBG*FINS. Blood total cholesterol (**f**) and blood triglycerides (**g**) were also analyzed to further characterize the lipid metabolism. n = 3 per group in the echocardiogram detection, n = 5–10 per group in the body weight and blood detection, * *P *< *0.05*, ** *P *< *0.01* vs. normal group

To evaluate the rat DCM model, normal and DCM rats were evaluated for biochemical indicators including fasting blood glucose, fasting serum insulin, blood total cholesterol and triglyceride levels, calculated insulin sensitivity indexes and echocardiography at 12 weeks. Echocardiography results showed that contractile function (LVEF, FS) and diastolic function (E/A ratio) were obviously decreased in the morbid rats (Fig. 2a). Additionally, the body weights of the DCM rats were significantly higher than those of the control rats after consuming the HFD, but at late stages of induced DCM, the weights of the DCM rats decreased gradually until they were lower than those of the normal rats (Fig. 2b). The hematological indicators FBG, FINS, TC and TG increased markedly in the DCM rats compared with the normal rats (Fig. 2c, d, f, g). The ISI calculated by FBG and FINS declined significantly after HFD and STZ treatment (Fig. 2e). These data are consistent with previous reports, thus indicating that the DCM model was successfully established in these rats [11].

### MSC infusion improved glucose and lipid metabolism, insulin resistance and cardiac function in DCM rats

**Fig. 3:**
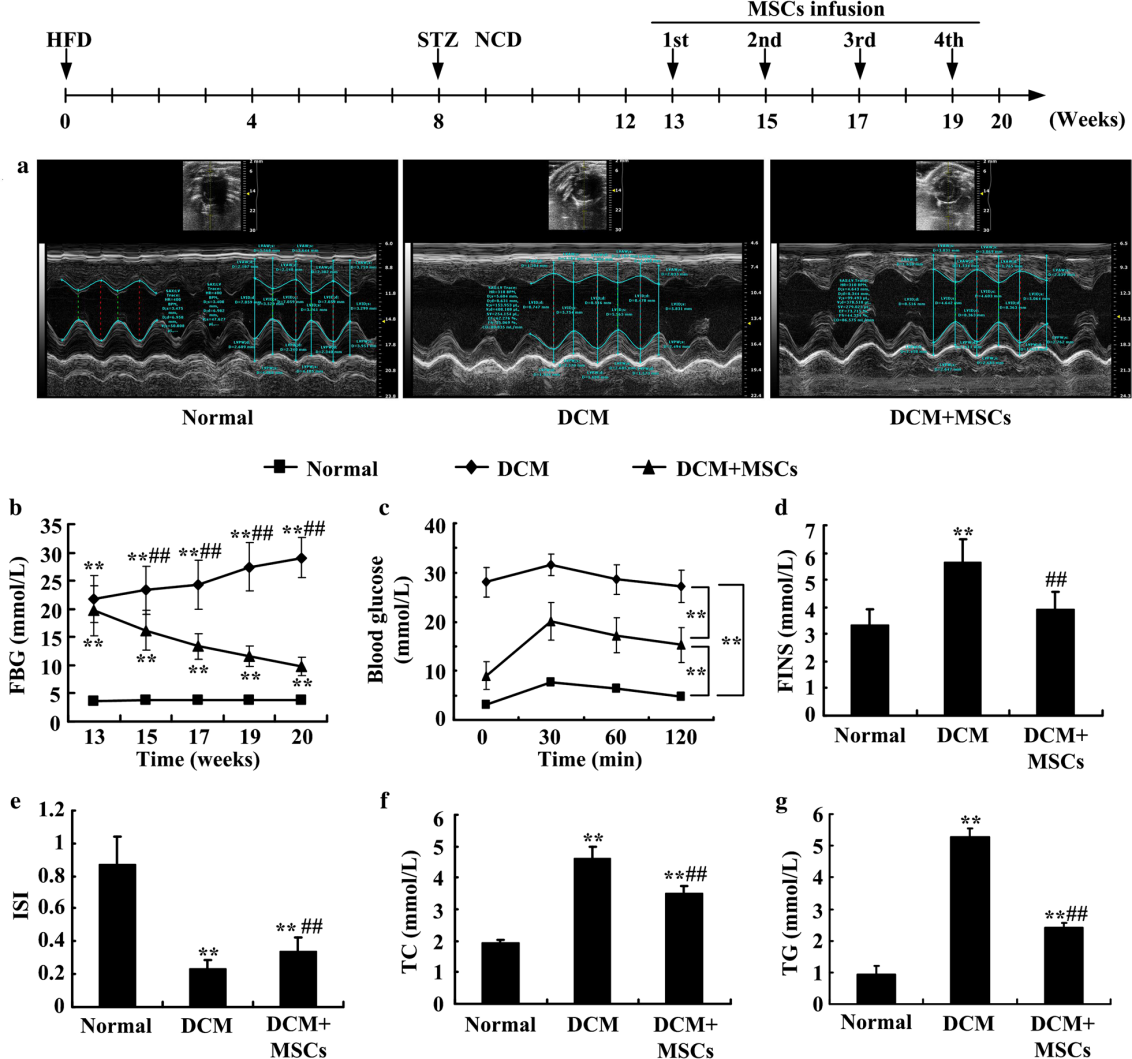
MSCs improved cardiac function, blood glucose and lipid metabolism in DCM rats. MSCs were infused 4 times, and cardiac function was measured by echocardiography (**a**) at 20 weeks. Fasting blood glucose (**b**) was assessed throughout the MSC infusion process. Intraperitoneal glucose tolerance testing (**c**), fasting serum insulin (**d**) and the insulin sensitivity index (**e**) were used to evaluate glucose metabolism. Blood total cholesterol (**f**) and blood triglycerides (**g**) were also analyzed to assess lipid metabolism at 19 weeks. n = 3–6 per group for echocardiographic assessment, n = 5–8 per group for hematological assessment, ** *P *< *0.01* vs. normal group, ## *P *< *0.01* vs. DCM group

To assess the effect of MSCs on glucose and lipid metabolism and cardiac function in DCM rats, we performed hematological assessments and echocardiography at 19 and 20 weeks, respectively. Echocardiography data (Fig. 3a, Table 1) demonstrated that the LVAWD, LVAWS, LVPWD and LVPWS of the DCM and MSC-treated groups were obviously thinner than those of the normal group. The LVIDD and LVIDS of the DCM and MSC-treated groups were significantly larger than those of the normal group. Compared with the DCM group, MSC administration alleviated ventricular wall thinning and ventricular chamber enlargement. The LVAWS in the MSC group was significantly thicker than that in the DCM group. LVEF and FS, which represent cardiac systolic function, were reduced in the DCM and MSC-treated rats, and MSCs slightly improved cardiac systolic function. The E’/A’ ratio, which reflects cardiac diastolic function, decreased dramatically in the DCM hearts. However, injecting MSC increased the E’/A’ ratio and ameliorated diastolic function significantly (Fig. 3a, Table 1). Blood sample results indicated that the fasting blood glucose levels of the DCM rats decreased significantly after MSC treatment (Fig. 3b). Moreover, as shown in Fig. 3c–g, glucose levels and lipid metabolism were improved in the MSC-treated rats. These data indicate that administering MSCs may improve diastolic function in DCM rats and partially ameliorate systolic function. In addition, MSC infusion may reverse hyperglycemia, blood lipid metabolic abnormalities and insulin resistance in DCM rats.

**Table 1:** Echocardiographic characteristics of rats at the end of experiment

	Normal	DCM	MSCs
LVAWD (mm)	2.359 ± 0.2079	1.270 ± 0.1633**	1.461 ± 0.1429**
LVAWS (mm)	3.900 ± 0.3437	2.180 ± 0.2090**	2.721 ± 0.2734**^,#^
LVIDD (mm)	6.682 ± 0.5339	8.206 ± 0.7136*	8.088 ± 0.6103*
LVIDS (mm)	2.980 ± 0.5424	5.055 ± 0.8686*	4.546 ± 0.5697*
LVPWD (mm)	2.462 ± 0.0453	1.316 ± 0.2192**	1.531 ± 0.1489**
LVPWS (mm)	4.073 ± 0.0453	2.309 ± 0.3865**	2.625 ± 0.2400*
LVEF (%)	85.043 ± 3.9506	66.729 ± 7.7492*	72.898 ± 6.4846
FS (%)	55.590 ± 4.5736	38.699 ± 6.2141*	43.811 ± 5.8114*
E’/A’	1.011 ± 0.0820	0.788 ± 0.1056*	0.954 ± 0.1056^#^

Data are presented as the mean ± SEM, n = 3–6 per group. LVAWD, LVAWS, LVPWD, LVPWS, LVIDD and LVIDS reflect ventricular structure and were measured at the lateral papillary muscle along the short axis. LVEF and FS represent ventricular contractile function and were calculated by systolic and diastolic indexes. E’/A’ reflects ventricular diastolic function and was captured by measuring mitral annular velocity. LVAWD: left ventricular end-diastolic anterior wall thickness; LVAWS: left ventricular end-systolic anterior wall thickness; LVIDD: left ventricular end-diastolic internal diameter; LVIDS: left ventricular end-systolic internal diameter; LVPWD: left ventricular end-diastolic posterior wall thickness; LVPWS: left ventricular end-systolic posterior wall thickness; LVEF: left ventricular ejection fraction; FS: fractional shortening; E’/A’: the ratio of the early to the late peak diastolic velocity. *P < 0.05, **P < *0.01* vs. normal Control; ^*#*^*P *< *0.05* vs. DCM

### MSC infusion ameliorated myocardial inflammation in DCM rats

**Fig. 4:**
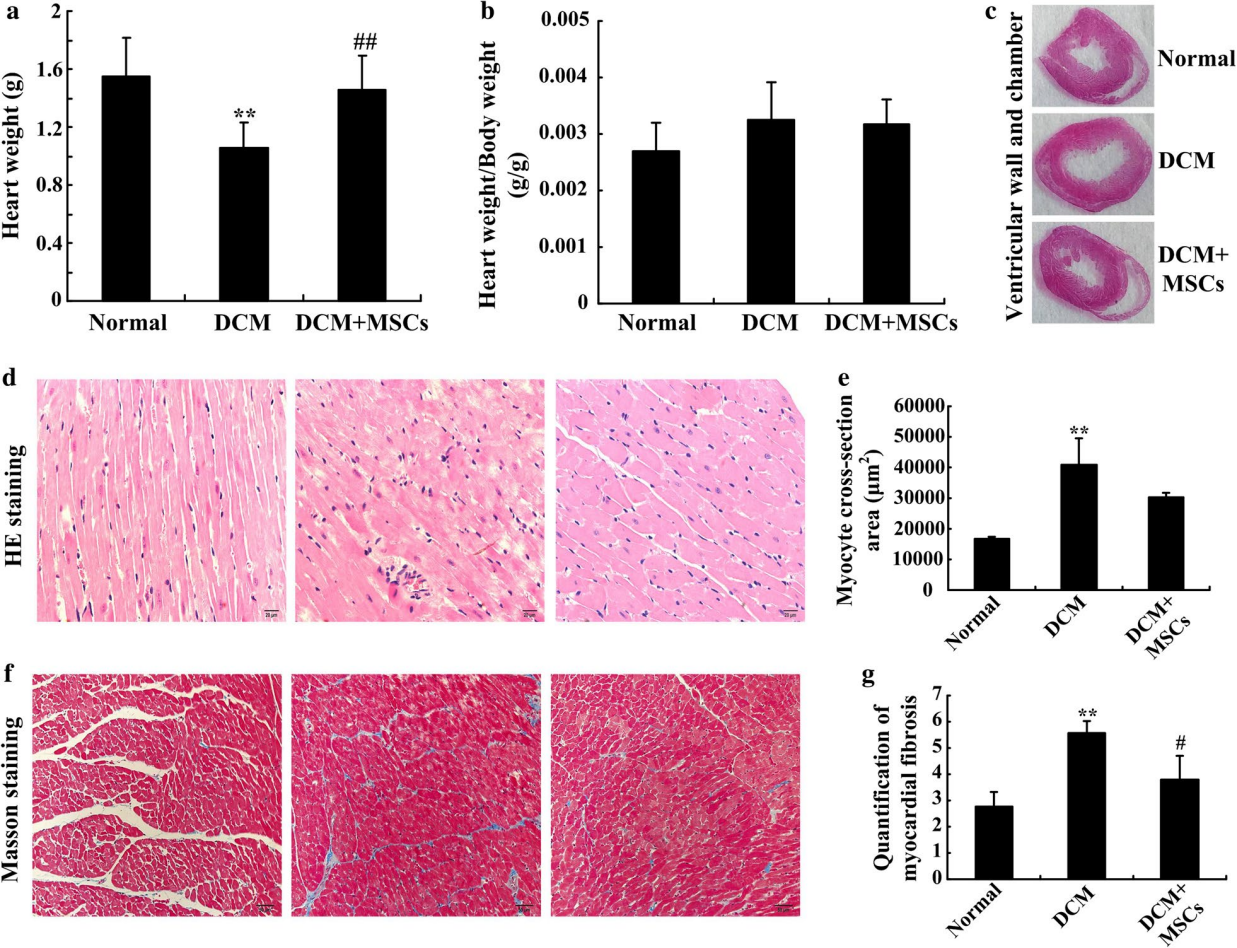
MSC infusion ameliorated abnormal metabolism, cardiac structure, inflammation and fibrosis in DCM rats. MSCs were infused 4 times, then rats were sacrificed, and their hearts were removed and weighed immediately (**a**), the ratio of heart weight to body weight (HW/BW) was calculated (**b**). Heart samples were fixed with paraformaldehyde, and cross sections of the ventricular wall and chamber were stained with H&E at the papillary muscle level (**c**). H&E staining (**d**, scale bar = 20 μm) was performed to reflect cardiomyocyte morphology and cardiac inflammation, and cardiomyocyte cross-sectional area (CSA) was also measured, analyzed and presented (**e**). Masson’s trichrome staining was performed to observe cardiac fibrosis (**f**, scale bar = 50 μm) and the quantification of myocardial fibrosis was also analyzed (**g**). n = 5–8 per group, **P < *0.01* vs. normal group, *#P *< *0.05*, *##P *< *0.01* vs. DCM group

To demonstrate the ability of MSCs to ameliorate inflammation and myocardial injury in DCM rats, we measured heart weight, calculated the ratio of heart weight to body weight (HW/BW) and stained heart sections with H&E and Masson’s trichrome. At the end of experiment, the heart weights of DCM rats were significantly lower than those of the normal rats (Fig. 4a), HW/BW of DCM group was larger than that of normal group (Fig. 4b). And staining the DCM hearts exhibited obvious myocardial thinning, chamber dilation, extensive myocardial disorganization, hypertrophic and atrophic cardiomyocytes, cell degeneration, inflammatory cell infiltration (Fig. 4c, d). Figure 4e showed that myocyte CSA of DCM hearts were significantly larger than that of normal hearts. Otherwise, Masson staining and quantitative analysis showed that DCM hearts existed abundant collagen deposition (Fig. 4f–g). However, the heart weights of the MSC-treated rats increased significantly and HW/BW was lower than that of DCM rats (Fig. 4a, b). Moreover, according to Fig. 4c, d, f, MSC-treated hearts presented decreased ventricular chamber, thicker wall, alleviated injury of myocardial and cardiomyocytes, reduced myocardial inflammation and interstitial fibrosis compared to DCM hearts. Myocyte CSA and quantification of myocardial fibrosis were also decreased in DCM + MSCs group (Fig. 4e, g). These data indicated that DCM rats had severely abnormal basal metabolism, abnormal myocardial structure and cardiomyocyte morphology, cardiac inflammation and interstitial fibrosis, all of which were ameliorated by MSC infusion.

### MSC infusion promoted M2 polarization in heart tissue and relieved chronic inflammation in DCM rats

**Fig. 5:**
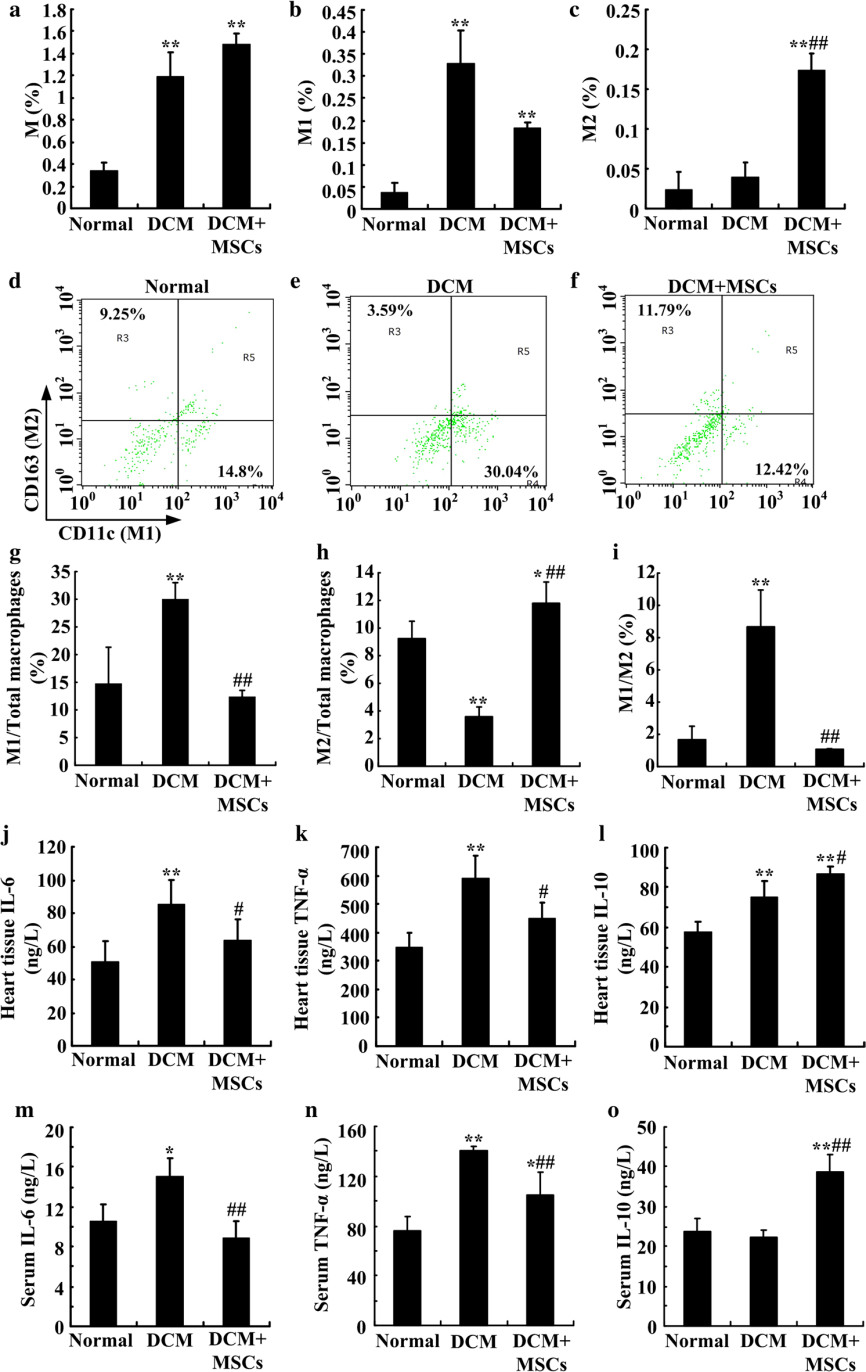
MSCs promoted M2 macrophage polarization in DCM hearts and relieved chronic inflammation in DCM rats. Heart tissues from the different groups were digested and stained with the cell surface markers CD68 (total macrophages), CD11c (M1), and CD163 (M2) and were analyzed by flow cytometry at 20 weeks. Ratios of total macrophages (**a**), M1 (**b**) and M2 (**c**) in total cells were analyzed. The percentage of M1 macrophages and M2 macrophages among CD68-positive total macrophages of normal (**d**, **g**), DCM (**e**, **h**) and DCM + MSCs (**f**, **i**) groups was presented and analyzed. IL-6, TNF-α and IL-10 concentrations in the heart homogenates (**j**, **k**, **l**) and blood serum (**m**, **n**, **o**) were detected by ELISA. n = 3–5 per group, *P < *0.05*, **P < *0.01* vs. normal group, *#P *< *0.05*, *##P *< *0.01* vs. DCM group

To analyze the influence of MSC treatment on macrophage polarization, we analyzed the phenotypes and ratios of cardiac macrophages by flow cytometry at 20 weeks. The results showed that the total number of cardiac macrophages increased significantly in both the DCM and MSC-treated rats (Fig. 5a). Notably, M1 macrophages among the total cells were predominant in the DCM rat hearts, while M2 macrophages were increased in the MSC-treated rats (Fig. 5b, c). Consistently, the proportion of M1 in total macrophages and M1/M2 of DCM hearts were significantly higher than those of normal and MSC-treated hearts. The percentage of M2 in total macrophages was decreased in DCM hearts, while it was increased obviously in MSC-treated hearts (Fig. 5d–f, g–i). There data demonstrated that increased macrophages in DCM hearts were M1 macrophages, and MSC treatment might increase the quantity and proportion of M2 macrophages significantly.

To further evaluate the MSC treatment effects, we analyzed chronic inflammation in the hearts and blood of the rats. As shown in Fig. 5, the proinflammatory cytokines IL-6 and TNF-α were significantly increased in the DCM group, whereas MSC treatment markedly reversed the cytokine elevation (Fig. 5j, k, m, n). Moreover, MSC treatment also significantly increased the anti-inflammatory cytokine IL-10 levels compared with those in the DCM group (Fig. 5l, o). These data indicated that M1 macrophages and proinflammatory cytokines play important roles in chronic and persistent inflammation in DCM rats. Furthermore, MSC infusion increased the M2 macrophage polarization, inhibited the proinflammatory response and increased anti-inflammatory cytokines to relieve inflammation in DCM hearts.

### MSCs promoted M2 macrophage polarization and alleviated inflammation in vitro

**Fig. 6:**
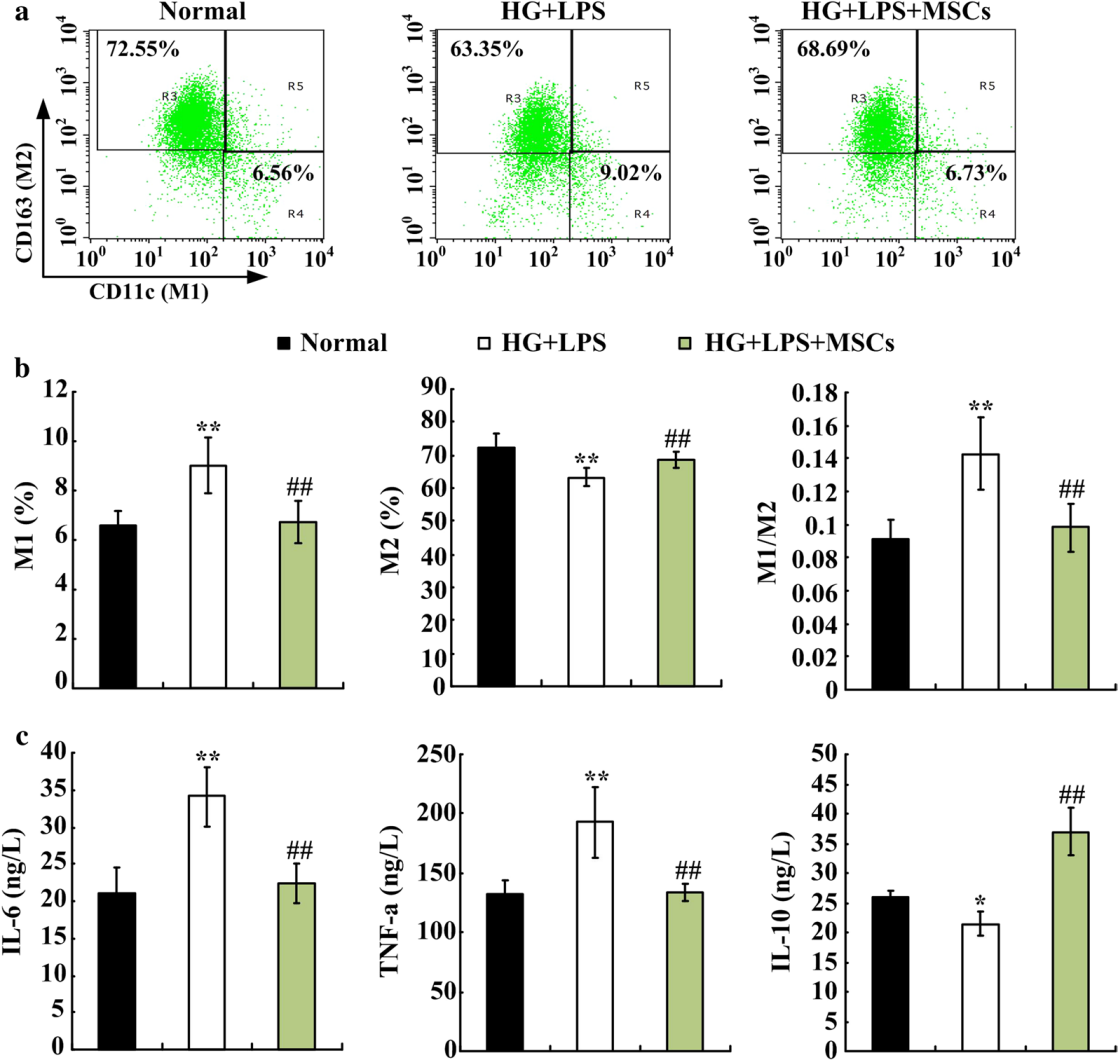
MSCs promoted M2 macrophage polarization and anti-inflammatory effects in high glucose conditions with LPS stimulation. Rat peritoneal macrophages were separated and divided into the normal, HG + LPS and HG + LPS + MSCs groups. After treatment, macrophages from the different groups were collected, and the M1 ratios, M2 ratios and the ratios of M1 to M2 phenotypes were detected by flow cytometry (**a**). The data were analyzed using Cellquest software (**b**). The IL-6, TNF-α and IL-10 concentrations in the supernatants were determined by ELISA (**c**). n = 4 per group. *P < 0.05, **P < *0.01* vs. normal group, *##P *< *0.01* vs. HG + LPS group

To further verify the effects of MSCs on ameliorating cardiac chronic inflammation via M2 macrophage polarization, we used an in vitro high-glucose (HG) and LPS-induced cell culture system to mimic the rat DCM model. Induced macrophages were cocultured with MSCs, and the ratios of different macrophage phenotypes were analyzed. The results showed that HG + LPS stimulation increased the number of CD11c-positive macrophages (M1) and reduced the number of CD163-positive macrophages (M2) compared with those of the normal group. Furthermore, the results indicated that coculturing with MSCs promoted polarization of M1 to M2 macrophages, as indicated by a significant increase in M2 macrophages ratios and a decrease in M1 macrophages ratios (Fig. 6a, b). Additionally, the secreted inflammatory cytokines, IL-6, IL-10 and TNF-α, were analyzed. The results showed that the IL-6 and TNF-α levels were significantly increased, but the IL-10 concentration significantly decreased in the HG + LPS group supernatant. Compared with the HG + LPS group, MSCs cocultured with macrophages inhibited IL-6 and TNF-α secretion and increased the IL-10 concentration (Fig. 6c). These data indicate that high glucose combined with LPS stimulation polarized macrophages to the M1 phenotype and stimulated the release of the proinflammatory cytokines IL-6 and TNF-α. MSCs could promote M2 macrophage polarization and stimulated IL-10 secretion.

### MSCs regulated M2 polarization via the COX-2-PGE2 pathway

**Fig. 7:**
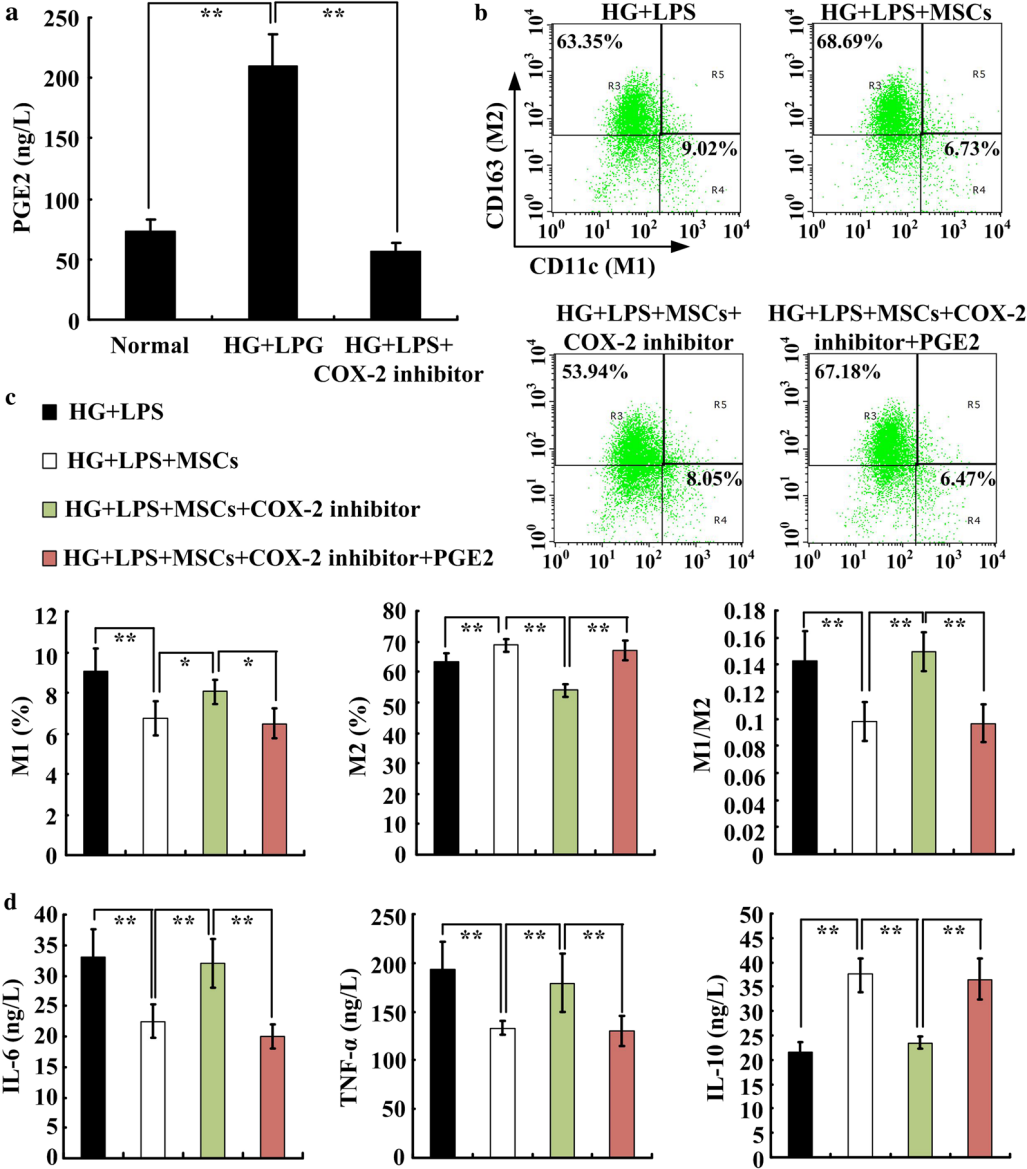
MSCs promoted M2 macrophage polarization and anti-inflammatory effects in an HG + LPS environment via the COX-2-PGE2 pathway. MSCs were incubated in normal control, HG + LPS or HG + LPS + COX-2-inhibitor media, and the PGE2 concentration in the supernatant was detected by ELISA (**a**). n = 4–5, **P < *0.01*. The HG + LPS-induced macrophages were cocultured with control MSCs or MSCs pretreated with COX-2 inhibitor in the presence or absence of exogenous PGE2. After treatment, macrophages were collected, and the M1 and M2 phenotypes were detected by flow cytometry (**b**, **c**). The IL-6, TNF-α and IL-10 concentrations in the supernatants were detected by ELISA (**d**). n = 3–4 per group, *P < 0.05, **P < *0.01*

Previous studies have demonstrated that PGE2 is involved in the immunoregulatory effects of MSCs [12]. Our results also showed a high concentration of PGE2 in the supernatant of MSCs stimulated with high glucose and LPS. Moreover, inhibiting COX-2 significantly decreased PGE2 secretion by MSCs (Fig. 7a). We further hypothesized that PGE2 might be involved in MSC-induced M2 macrophage polarization. To verify this, we pretreated MSCs with 10 μM COX-2 inhibitor to specifically suppress PGE2 expression, then analyzed the macrophage polarization. The results showed that the M1 macrophages increased significantly in the COX-2-inhibited group, whereas the M2 macrophages decreased significantly compared with those in the MSC group. Exogenous PGE2 was then added to the COX-2-inhibited group, and the M1 and M2 macrophage polarizations were significantly reversed (Fig. 7b, c). Moreover, the IL-6 and TNF-α levels were significantly increased, but the IL-10 level was significantly decreased in the COX-2-inhibited group. Adding exogenous PGE2 also inhibited the IL-6 and TNF-α levels and increased IL-10 levels (Fig. 7d). These results indicated that MSCs might promote M2 macrophage polarization by secreting PGE2, which can alleviate the chronic inflammation induced by high glucose and LPS.

## Discussion

Long-term uncontrolled high blood glucose can result in microvascular and macrovascular disorders that lead to severe complications in vital organs such as the heart, brain and kidney, resulting in high morbidity and mortality. Diabetic cardiomyopathy (DCM) is a common complication of diabetes and is an important factor that contributes to the mortality of diabetic patients [13]. The pathophysiological mechanisms of DCM involve uncontrolled high glucose levels, advanced glycation end products (AGEs), insulin resistance, overabundance of free fatty acids, and triglycerides [2, 14]. Moreover, peroxisome proliferator-activated receptor (PPARα) induced reactive oxygen species (ROS) is also involved, especially in type 2 diabetes [2]. These pathophysiological processes induce a vicious cycle that exacerbates cytotoxicity and inflammatory cytokine release, resulting in chronic inflammation [2, 15]. This chronic inflammation is known as a metabolic-related inflammatory response, which impacts cardiac function by promoting myocardial cell death and interstitial fibrosis and so on [16, 17]. However, no effective treatment exists to prevent the development and aggravation of this metabolic-related inflammation in DCM.

Previous studies have demonstrated that macrophages play important roles in regulating the inflammatory response [4]. In different microenvironments, macrophages are polarized to different phenotypes and exert different functions [4, 5]. The two major macrophage phenotypes include classically activated macrophages (M1) and alternatively activated macrophages (M2). M1 macrophages secrete proinflammatory cytokines and chemokines, aggravating inflammation and myocardial injury [18, 19]. In contrast, M2 macrophages are reparative and exert anti-inflammatory effects [20]. Thus, increasing the quantity or ratio of M2 macrophages in injured regions is an attractive treatment. This potential treatment has attracted much attention, and researchers have begun seeking potential candidates to regulate macrophage polarization [21, 22]. Our previous study found that MSC infusion promoted macrophage polarization to the M2 phenotype in insulin-sensitive tissues and improved insulin sensitivity and alleviated hyperglycemia in type 2 diabetic rats [23]. While DCM is considered a major complication of diabetes, the MSC infusion’s role in macrophage polarization in DCM remains unknown.

In our present study, MSC infusion reversed abnormal FBG, FINS, TC and TG levels in the blood of DCM rats. These abnormal biochemical indicators were considered major risk factors for DCM. Moreover, the echocardiogram results indicated that MSC infusion ameliorated ventricular wall thinning, ventricular chamber dilation and cardiac diastolic and systolic functions. The histology results indicated that MSC-treated rats had improved heart weight and structure and that MSC infusion alleviated cardiomyocyte hypertrophy, inflammation and collagen deposition in DCM hearts. These results demonstrated that MSC infusion can alleviate metabolic disorders and cardiac injury in DCM rats. However, the underlying mechanisms require further study.

In DCM heart tissue, diabetes mellitus-induced chronic inflammation is a pathophysiological process that induces the release of inflammatory cytokines and ultimately aggravates myocardial injury [2]. Because macrophages have important roles in regulating inflammation, we analyzed the ratios of cardiac macrophage phenotypes. The results showed that the total number of macrophages increased significantly and included predominantly M1 macrophages in the DCM rat hearts. In addition to M1 macrophage polarization, the classic proinflammatory cytokines TNF-α and IL-6 were elevated in both the blood and the heart, and anti-inflammatory cytokine IL-10 levels were inhibited, especially in the blood. Previous reports showed that along with cardiac injury, circulating monocytes are recruited to the injured myocardium to replace resident macrophages (mainly M2) and then differentiate into inflammatory M1 macrophages and aggravate myocardial injury [24, 25]. Accordingly, a novel and potent strategy to ameliorate inflammation and myocardial injury may be to upregulate the quantity and proportion of resident M2 macrophages or to promote M2 macrophage renewal and proliferation. In our study, MSC infusion significantly increased the M2 macrophage polarization and reversed the proinflammatory cytokine elevation and the anti-inflammatory cytokine inhibition in the blood and heart. These results suggested that different macrophage phenotypes play important roles in the development and treatment of DCM and that MSC infusion can promote polarization of M1 to M2 macrophages in DCM hearts.

We additionally investigated the underlying mechanism of MSCs in regulating macrophage polarization in vitro. A previous study demonstrated that PGE2 is a key modulator of MSC-mediated inflammation in a sepsis model [12]. In our study, high glucose was administered with LPS stimulation in vitro to mimic the DCM microenvironment. We found that high glucose combined with LPS stimulated MSCs to secrete large amounts of PGE2. Moreover, high glucose combined with LPS induced M1 macrophage polarization to release proinflammatory cytokines. Similar to our in vivo results, coculturing with MSCs increased the proportion of M2 macrophages and secretion of anti-inflammatory cytokines in vitro. To further confirm that PGE2 was involved in the MSC-mediated M2 macrophage polarization, we used a COX-2 inhibitor, which is a key oxidase inhibitor during PGE2 stimulation [12, 26]. Inhibiting COX-2 significantly decreased PGE2 levels in the supernatants of high-glucose and LPS-induced MSCs. Moreover, in the in vitro DCM model, coculturing MSCs that were pretreated with a COX-2 inhibitor with macrophages failed to reverse the M1 macrophage polarization and increased the pro-inflammatory cytokines. However, these effects were restored by adding exogenous PGE2 to the coculture system. These results suggest that the COX-2-PGE2 pathway might be involved in MSC-mediated M2 macrophage polarization in DCM injury.

## Conclusion

In summary, our results demonstrated that MSC infusion improved glucose and lipid metabolism and cardiac structure and function in DCM rats. Moreover, MSCs can alleviate myocardial inflammation and interstitial fibrosis partly via COX-2-PGE2-mediated polarization of M2 macrophages and IL-10 secretion in DCM hearts. These findings may provide a novel and efficient measure for treating diabetic cardiomyopathy.

## Data Availability

Data and study materials are available.

## Abbreviations

DCM: diabetic cardiomyopathy
AD-MSCs: adipose-derived mesenchymal stem cells
STZ: streptozotocin
LPS: lipopolysaccharide
COX-2: cydooxygenase-2
PGE2: prostaglandin E2
FBS: fetal bovine serum
HFD: high-fat diet
FBG: fasting blood glucose
FINS: fasting insulin
ISI: insulin sensitivity index
TC: total cholesterol
TG: triglyceride
IPGTT: intraperitoneal glucose tolerance test
ELISA: enzyme-linked immunosorbent assay
FS: fractional shortening
LVEF: left ventricular ejection fraction
E’/A’: the ratio of early to late peak diastolic velocity
IL-6: interleukin-6
IL-10: interleukin-10
TNF-α: tumor necrosis factor-α
